# Automatic bundle-specific white matter fiber tracking tool using diffusion tensor imaging data: A pilot trial in the application of language-related glioma resection

**DOI:** 10.3389/fonc.2023.1089923

**Published:** 2023-03-24

**Authors:** Yifan Yuan, Tianming Qiu, Shin Tai Chong, Sanford Pin-Chuan Hsu, Ying-Hua Chu, Yi-Cheng Hsu, Geng Xu, Yu-Ting Ko, Kuan-Tsen Kuo, Zixiao Yang, Wei Zhu, Ching-Po Lin, Jianping Song

**Affiliations:** ^1^ Department of Neurosurgery, Huashan Hospital, Shanghai Medical College, Fudan University, Shanghai, China; ^2^ National Center for Neurological Disorders, Shanghai, China; ^3^ Neurosurgical Institute of Fudan University, Shanghai, China; ^4^ Shanghai Clinical Medical Center of Neurosurgery, Shanghai, China; ^5^ Shanghai Key Laboratory of Brain Function Restoration and Neural Regeneration, Shanghai, China; ^6^ Research Units of New Technologies of Micro-Endoscopy Combination in Skull Base Surgery, Chinese Academy of Medical Sciences (CAMS), Shanghai, China; ^7^ Institute of Neuroscience, National Yang Ming Chiao Tung University, Hsinchu, Taiwan; ^8^ Department of Neurosurgery, Neurological Institute, Taipei Veterans General Hospital, Taipei, Taiwan; ^9^ Magnetic Resonance (MR) Collaboration, Siemens Healthineers Ltd., Shanghai, China; ^10^ Department of Neurosurgery, National Regional Medical Center, Fudan University Huashan Hospital, Fuzhou, Fujian, China

**Keywords:** awake neurosurgery, brain mapping, diffusion tensor imaging, functional neuroimaging, white matter tracts fiber tracking software for neurosurgeon

## Abstract

Cerebral neoplasms like gliomas may cause intracranial pressure increasing, neural tract deviation, infiltration, or destruction in peritumoral areas, leading to neuro-functional deficits. Novel tracking technology, such as DTI, can objectively reveal and visualize three-dimensional white matter trajectories; in combination with intraoperative navigation, it can help achieve maximum resection whilst minimizing neurological deficit. Since the reconstruction of DTI raw data largely relies on the technical engineering and anatomical experience of the operator; it is time-consuming and prone to operator-induced bias. Here, we develop new user-friendly software to automatically segment and reconstruct functionally active areas to facilitate precise surgery. In this pilot trial, we used an in-house developed software (DiffusionGo) specially designed for neurosurgeons, which integrated a reliable diffusion-weighted image (DWI) preprocessing pipeline that embedded several functionalities from software packages of FSL, MRtrix3, and ANTs. The preprocessing pipeline is as follows: 1. DWI denoising, 2. Gibbs-ringing removing, 3. Susceptibility distortion correction (process if opposite polarity data were acquired), 4. Eddy current and motion correction, and 5. Bias correction. Then, this fully automatic multiple assigned criteria algorithms for fiber tracking were used to achieve easy modeling and assist precision surgery. We demonstrated the application with three language-related cases in three different centers, including a left frontal, a left temporal, and a left frontal-temporal glioma, to achieve a favorable surgical outcome with language function preservation or recovery. The DTI tracking result using DiffusionGo showed robust consistency with direct cortical stimulation (DCS) finding. We believe that this fully automatic processing pipeline provides the neurosurgeon with a solution that may reduce time costs and operating errors and improve care quality and surgical procedure quality across different neurosurgical centers.

## 1 Introduction

Glioma is the most common malignant intradural tumor, with a new incidence of about 4 per 100,000 people worldwide every year (1). Surgery is the first-line treatment for debulking tumors and obtaining tissues for pathology analysis. It has widely been agreed that the extent of tumor removal is positively correlated with patient survival and that residues surrounding the tumor margin always lead to early recurrence (2, 3). However, glioma grows infiltratively along fiber tracts, making it difficult to determine the tumor boundary only according to surgeons’ experiences. Extended resection may impair eloquent brain areas and cause functional disorders such as hemiplegia and aphasia. Therefore, precise tracing of tumor boundary is the key to balancing the survival and quality of life of glioma patients (4).

Diffusion tensor imaging (DTI) is a noninvasive technique that can probe the molecular diffusivity of water within the white matter to reflect the intravoxel architecture by measuring the water self-diffusion tensor (5, 6). Linking the anisotropic orientation determined by the principal eigenvector of the tensor has been widely applied to map neuronal tracts (7, 8). This method has been used to reveal and visualize three-dimensional white matter trajectories and provides crucial information to neurosurgeons for neurosurgical planning and navigation (9, 10). Thus, DTI tractography has been considered routine for many neurosurgical procedures.

Many imaging techniques and surgical adjuncts, such as integrated neuro-navigation with DTI or blood oxygen level-dependent functional magnetic resonance imaging (BOLD-fMRI), have been developed to delineate the tumor margin and protect eloquent areas to avoid increasing postoperative deficits during aggressive tumor resection (11). Currently, these techniques are routinely applied in many neurosurgical procedures for cortical eloquence and white matter assessment. However, the complexity of imaging processing will increase the clinical burden, and insufficient experience in imaging processing in some clinical centers may misguide the surgical procedure, leading to consequent complications. Therefore, a simple, automatic, less time-consuming, high-accuracy imaging processing and easy-to use software is needed to reduce the clinical burden for the neurosurgeon and narrow the gap between different clinical centers.

Imaging quality and preprocessing procedures are crucial to obtain robust and reliable tractography for neurosurgery. Despite having a few helpful workstations provided by the magnetic resonance imaging (MRI) machine vendors and powerful software (such as MRtrix3, FSL, DSI-studio, 3D-Slicer, etc.) used for reconstructing neural tractography, complicated processing procedures, and reconstructing reliability have hindered its clinical applications, especially for neurosurgery (12). A well-trained technician or surgeon must integrate different software programs for surgical planning based on prior anatomical knowledge, which is time-consuming and prone to operator-induced bias (13). Thus, an automatic imaging processing pipeline and fiber tractography segmentation tool are necessary.

Here, we develop an in-house software, “DiffusionGo” that integrates a fully automatic preprocessing pipeline for diffusion MRI data and a boosted tractography algorithm to achieve efficient and reliable modeling. In this pilot trial, the surgical plan of three language-related cases from multicenter, including one left frontal-temporal-insular glioma, one left temporal glioma, and one left frontal-insular glioma, was reconstructed by DiffusionGo, and favorable surgical outcomes with language function preservation was achieved.

## 2 Materials and methods

### 2.1 Study design and patient recruitment

This was a multicenter, observational, prospective pilot trial. We report three patients with preoperatively imaging-diagnosed gliomas who underwent surgical resection at Huashan Hospital (Shanghai, China), Taipei Veterans General Hospital (Taipei, Taiwan), and the First Affiliated Hospital of Fujian Medical University (Fujian, China) between December 2020 and March 2022. We collected clinical, imaging, treatment, and outcome data. This study was approved by the local Hospital Ethics Committee (Approval No. KY2021-452 and KY2019-008) and was conducted under the Declaration of Helsinki. All patients were verbally informed, and the signature of a specific informed consent was obtained.

### 2.2 Imaging acquisition

As a pilot trial, three typical cases from three different centers, respectively, with completed MRI and clinical assessment, were described here. The MRI images of the first case were acquired on a 3.0 Tesla MRI scanner (Magnetom Verio; Siemens, Erlangen, Germany) with a 12-channel head coil at Huashan Hospital, Shanghai, China, including high-resolution three-dimensional T1-weighted images (T1WI, TR = 1630 ms; TE = 2.9 ms; flip angle = 9°; field of view (FOV) = 172 x 250 x 176 mm^3^; voxel size = 1 x 1 x 1 mm^3^), diffusion-weighted images (DWI, TR = 6500 ms; TE = 95 ms; FOV = 220 x 220 x 140 mm^3^; voxel size = 2 x 2 x 2 mm^3^, 30 directions of b value = 1000 s/mm^2^; average = 2).

The MRI images of the second case were acquired on a 3.0 Tesla MRI scanner (Siemens Magnetom Tim Trio, Erlangen, Germany) at National Yang Ming Chiao Tung University, Taipei, Taiwan, using a 12-channel head array coil. High-resolution T1W images were acquired using a 3D magnetization-prepared rapid gradient echo sequence (MPRAGE, TR/TE = 2530/3.5 ms; TI = 1100 ms; FOV = 256 mm; voxel size = 1 x1 x 1 mm^3^; flip angle = 7°) for image segmentation, registration, and brain mask extraction. Multishelled, multiband DWIs were acquired using a single-shot spin-echo planar imaging sequence (monopolar scheme; TR = 3525 ms; TE = 109.2 ms; FOV = 240 x 240 x 144 mm^3^; voxel size: 2 × 2 × 2 mm^3^; multiband factor = 3; phase encoding: anterior to posterior) with two b-values of 1000 s/mm^2^ (30 diffusion directions) and 3000 s/mm^2^ (60 diffusion directions), in which b0 images were interleaved in every six volumes. Data with the same DWI protocol using an opposite polarity (phase encoding from posterior to anterior) were also acquired for three B_0_ images.

The MRI images of the third case were acquired on a 3.0 Tesla MRI scanner (Siemens Magnetom Prisma, Erlangen, Germany) at the First Affiliated Hospital of Fujian Medical University, Fuzhou, Fujian, China, using a 64-channel head array coil. High-resolution T1W images with a 3D MPRAGE sequence (TR/TE = 2300/2.32 ms; TI = 946 ms; FOV = 240 mm; voxel size = 0.94 x0.94 x 0.90 mm^3^; flip angle = 8) and DWI with a single shot spin-echo planar imaging sequence (monopolar scheme; TR = 3700 ms; TE = 92 ms; FOV = 220 x 220 x 130 mm^3^; voxel size: 1.72 × 1.72 × 5.2 mm^3^ with 20 diffusion directions and b-values of 1000 s/mm^2^) were acquired.

### 2.3 Automatic bundle-specific neuro-fiber tractography by DiffusionGo

**Figure 1:**
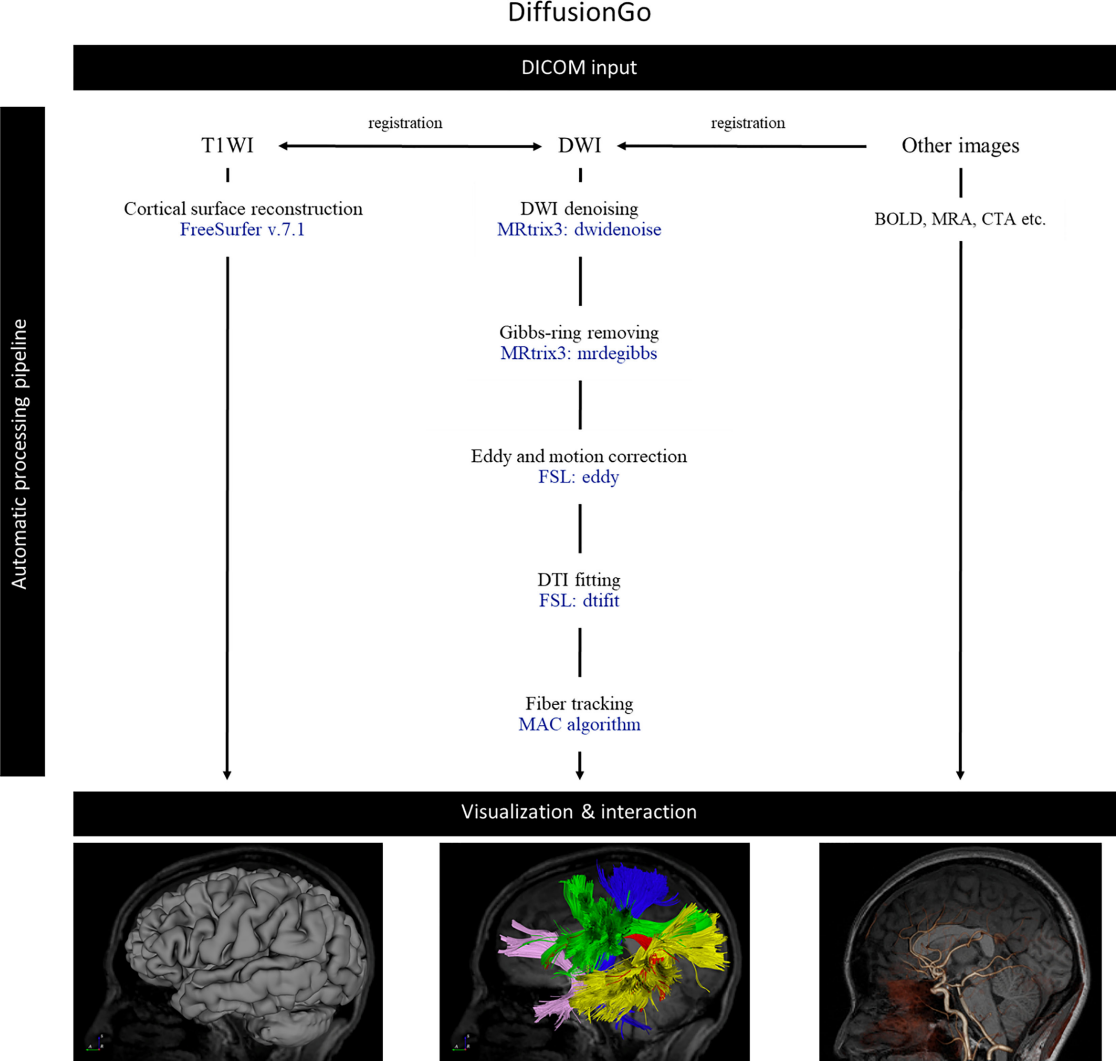
The automatic pipeline of DiffusionGo. MAC, multiple assigned criteria; DICOM, digital imaging and communications in medicine; DSA, digital subtraction angiography; DWI, diffusion weighted image; DTI, diffusion tensor imaging; BOLD, blood oxygen level-dependent.

DiffusionGo integrates a reliable preprocessing pipeline with a fully automatic multiple assigned criteria algorithm for bundle-specific tractography using DTI data based on anatomical connectivity (14). First, structural (typo) images were coregistered with DWI by using Advanced Normalization Tools (ANTs, http://stnava.github.io/ANTs/). All DWIs underwent diffusion preprocessing pipeline and DTI model fitting with MRtrix3 (https://www.mrtrix.org) (15) and FSL (https://fsl.fmrib.ox.ac.uk/fsl/fslwiki) (16): 1. DWI denoising (17–19), 2. Gibbs-ringing removing (20), 3. Susceptibility distortion correction (process if opposite polarity data were acquired), 4. Eddy current and motion correction (21), 5. Bias correction (22), and 6. DTI fitting. A patent-protected multiple assigned criteria (MAC) algorithm (14) for fiber tracking was used. The motor pathway (corticospinal tract, CST), language pathway (arcuate fasciculus, AF, superior longitudinal fasciculus, SLF, frontal aslant tract, FAT, inferior longitudinal fasciculus, ILF, inferior fronto-occipital fasciculus, IFOF, and uncinate fasciculus, UF), and visual pathway (optic radiation, OR) were segmented automatically. The potential false-positive tracts were identified and manually removed by experiences neurosurgeon. The workflow is demonstrated in **Figure 1**. Validation results for the DiffusionGo automatic fiber tractography are summarised in the **Supplementary Material**.

### 2.4 Three-dimensional visualization

The cortical surface was reconstructed by FreeSurfer (version 7.1, https://surfer.nmr.mgh.harvard.edu) (23) and integrated into DiffusionGo with DTI tractography to build 3D model visualization.

## 3 Case study

**Figure 2:**
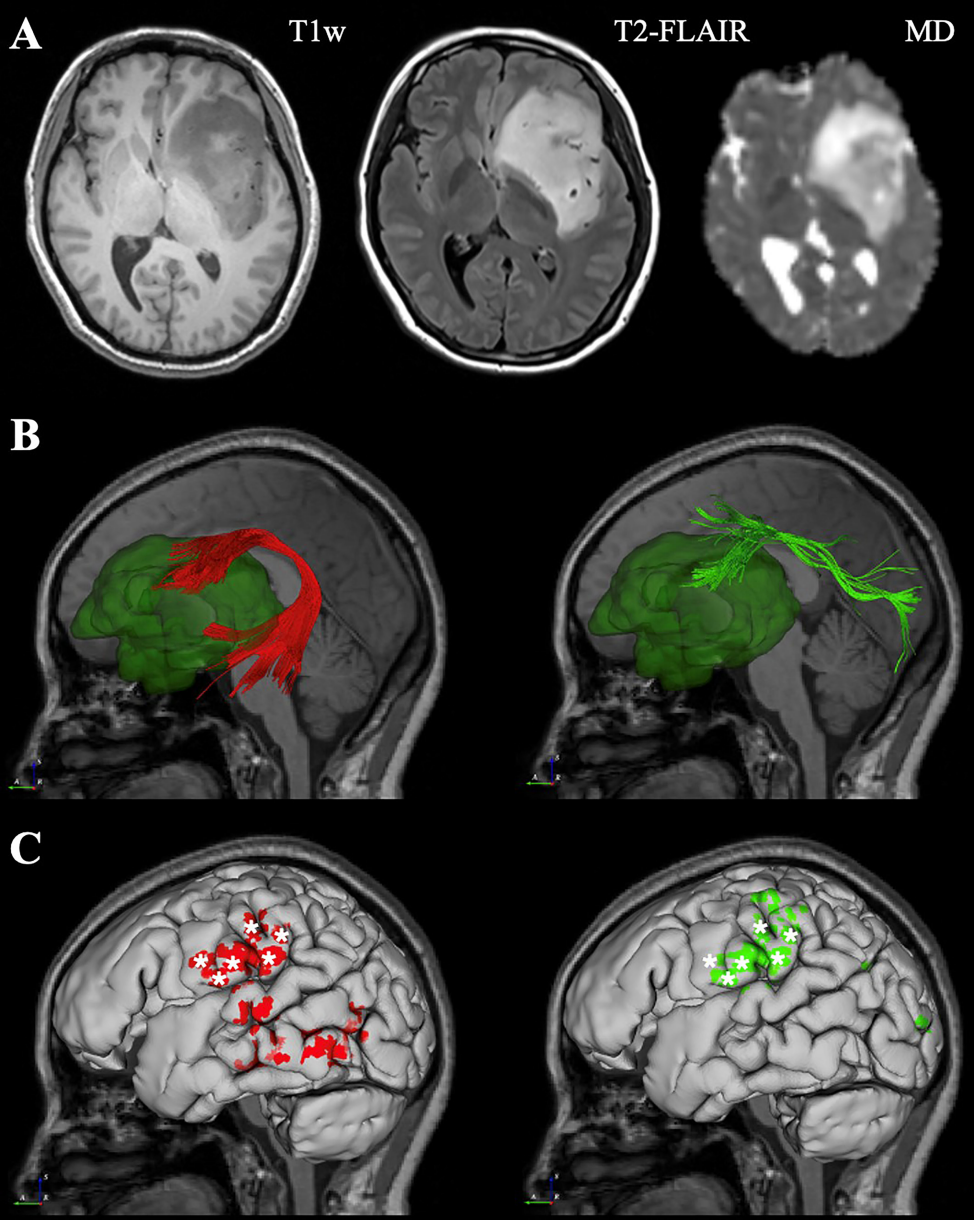
Representive imaging of a patient with left frontal-temporal lobe astrocytoma. Case 1, a 42-year-old woman with left frontal-temporal lobe astrocytoma (WHO grade II) in MR images **(A)**. The relationship between language-related fiber tracts (AF in red and SLF-II in green color) and tumor (dark green color) were shown in **(B)**. The cortical termination of each tract was projected on the cortical surface **(C)**. The eloquent area of intro-operative speech arrest (DCS) was marked with stars. (MD, Mean Diffusivity).

Here, three exemplary cases from three different neurosurgical centers were demonstrated respectively. The first case was a 42-year-old woman with left frontal-temporal-insular lobe astrocytoma in Huashan Hospital. The lesion was about 79mm in diameter with high signal in T2 and not enhanced after contrast; and had a close relationship to the speech output language area with high surgical risk. Considering the tumor might not be highly aggressive and malignant and was more sensitive to subsequent radiotherapy and chemotherapy, we focused more on functional protection to maintain a relatively high living quality for the patient. DTI fiber tractography and conventional MRI sequences were integrated with DiffusionGo for surgical planning (**Figure 2**). Multimodality-guided awake surgery under electrophysiology monitoring for language function mapping and preservation was used. Speech arrest was defined as discontinuing number counting without simultaneous motor response by direct cortical stimulation (DCS). In the language mapping phase, we found that the eloquent area of speech arrest was located in the classical Broca’s area as the terminal territory of our reconstructed AF. The surgery was conducted under awake surgery, and the tumor subtotally resected. There was no language dysfunction during the whole procedure. The patients was finally diagnosed as astrocytoma, WHO grade 2, IDH mutant. For this case, the automatic algorithm was used to reconstruct the AF and SLF-II, which were considered the major white matter tracts adjacent to the lesion.

**Figure 3:**
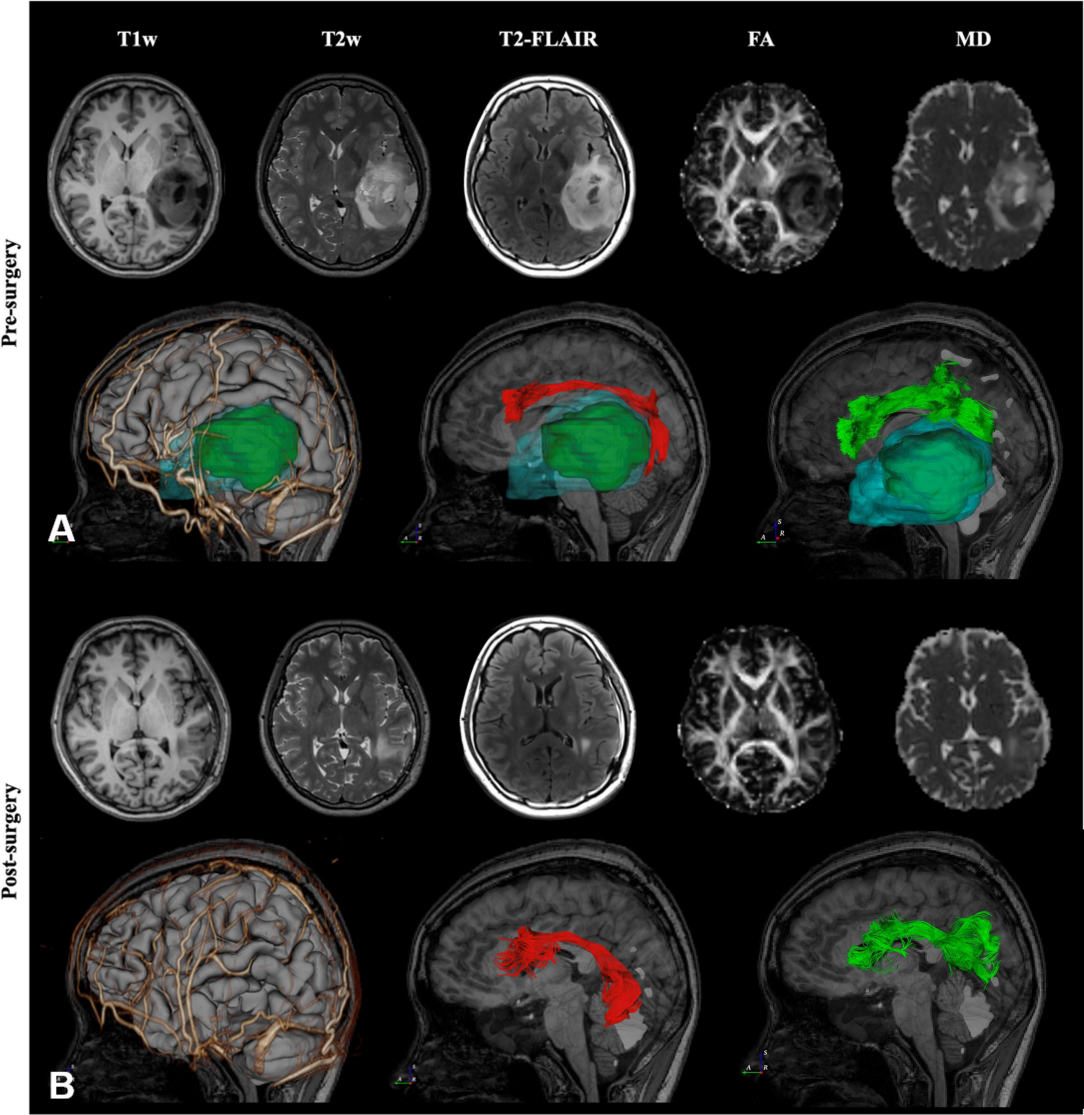
Preoperative and post-operative imaging of a patient with left temporal lobe astrocytoma. Case 2, A 41-year-old woman with temporal lobe glioblastoma over the left temporal lobe with mild perifocal edema was reconstructed and displayed in DiffusionGo **(A)**. The AF (red) and SLF-II (green) were automatically reconstructed and integrated with the cortical surface (gray), arteries and veins (gold), tumor (dark green), and peritumoral edema (light blue with translucent). Superior displacement of the left Wernicke’s area was identified with intact AF projecting to the left premotor and left Broca’s areas. After a complete survey, since the patient could not endure an awake surgery, tumor removal was performed under general anesthesia without any neurological deficits postoperatively. Two months later, MRI of the brain revealed total gross removal without residual tumor, and DTI showed preservation of the AF (red) and SLF-II (green) **(B)**. (FA, Fractional Anisotropy).

The second case was diagnosed and treated at Taipei Veterans General Hospital. This 41-year-old woman suffered from intermittent headaches, which progressed gradually. In addition, she could not write or read words that she knew. Ignoring the objects on her right side was also noted. She went to the clinic, and MRI of the brain showed a heterogeneous mass, 55 mm in diameter, over the left temporal lobe with mild perifocal edema. DTI fiber tractography of the language-related pathways (AF, and SLF-II) was reconstructed and displayed in DiffusionGo. Superior displacement of the left Wernicke’s area was identified with intact AF projecting to the left premotor and left Broca areas (**Figure 3**). After a complete survey, since the patient could not endure an awake surgery, tumor removal was performed under general anesthesia without any neurological deficits postoperatively. MRI of the brain revealed total gross removal without residual tumor. Unfortunately, glioblastoma was diagnosed.

**Figure 4:**
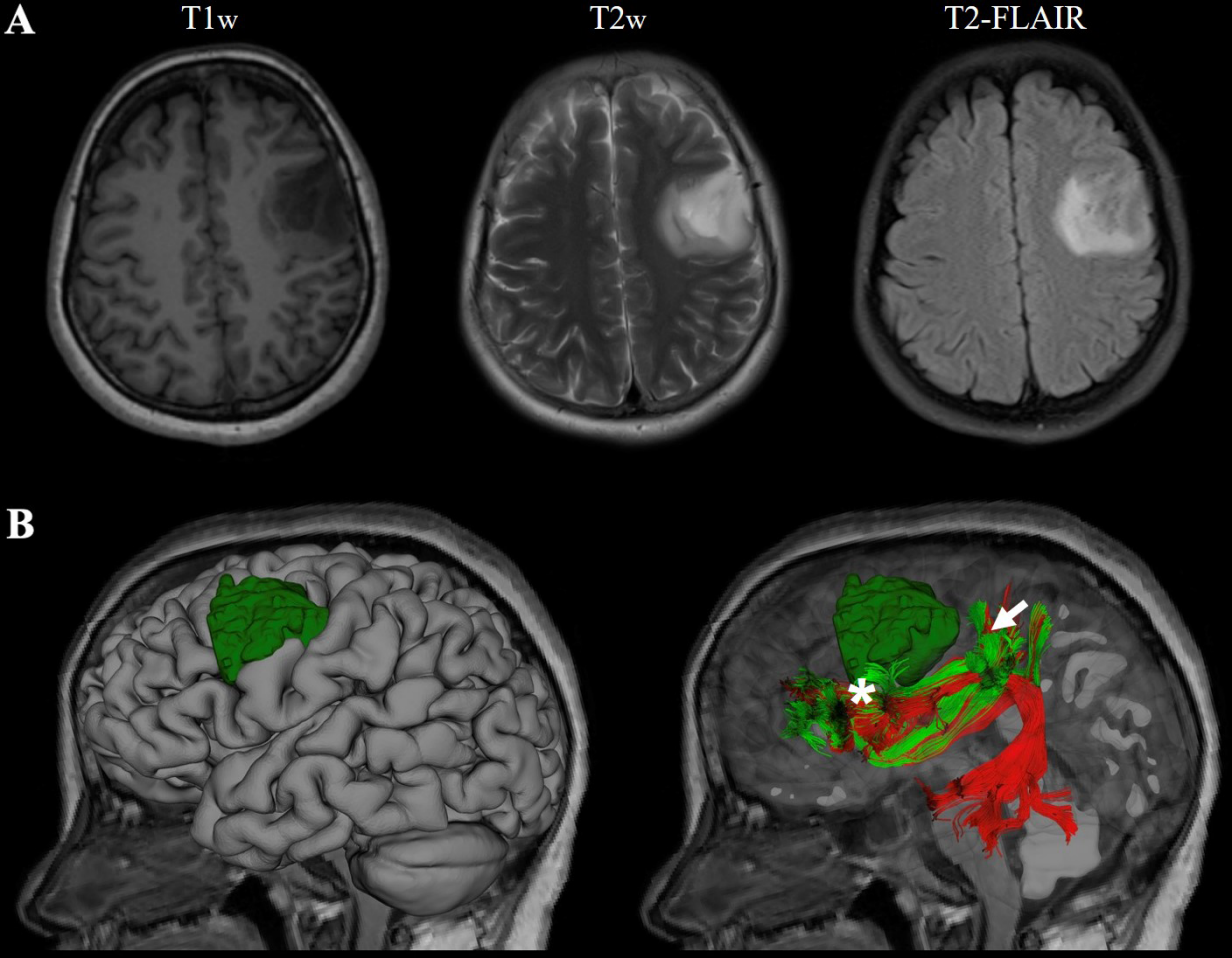
Representive imaging of a patient with left frontal lobe astrocytoma. Case 3, a 41-year-old female with left frontal lobe astrocytoma (WHO grade III) in MR images **(A)**. The relationship between language-related fiber tracts (AF in red and SLF-II in green color) and tumor (dark green) were shown in **(B)**; the eloquent area of intro-operative speech arrest (DCS) was marked with a star(Projecting terminal territory of AF) and an arrow (Projecting terminal territory of SLF-II).

The third case was recently conducted at the First Affiliated Hospital of Fujian Medical University. This 41-year-old female suffered from recurrent seizures attack for 4 years. Conventional MR indicates a left frontal-insular lesion of 3.8 cm, with high signal in T2WI and not enhanced after contrast. Preoperative DTI showed the AF and SLF were located below and behind the tumor, respectively (**Figure 4**). Considering the close relationship between the lesion and Broca’s region, multimodality-guided awake surgery under electrophysiology monitoring for language function mapping and preservation was conducted. After craniotomy, in the language mapping phase using DCS, we found that the eloquent area of speech arrest was located in the terminal territory of our reconstructed AF and SLF. The surgery was conducted under awake surgery. Since there was no clear boundary between the tumor and the eloquent area and SLF behind the tumor, a subtotal resection of tumor was achieved. There was no language dysfunction during the whole procedure. The pathology showed anaplastic astrocytoma, and the patient was discharged for adjuvant radiotherapy.

## 4 Discussion

Sensorimotor and language eloquence are considered higher brain function, and even mild impairment causes poor outcomes (24). Unlike the sensorimotor area, the eloquent language area is more diverse, and currently, researchers have revealed that it is quite different from classical canonical classification (25, 26). Classic language eloquent models posited that motor and sensory language cortex existed in Broca’s area (including pars triangularis and pars opercularis) and Wernicke’s area, respectively (26). However, cortical maps generated with intraoperative direct cortical stimulation (DCS) data revealed extraordinary variability in language localization in the dominant hemisphere, and the eloquent language areas are quite different from the classical canonical models (27). The expression tasks are best mapped in the frontal lobe, with 100% of sensitivity and 66% of specificity with a 5-mm resolution (28). Tate et al. found that speech arrest regions (or the speech output region) seemed to be localized in the ventral premotor cortex rather than the classical Broca’s area (29). According to Wu et al. study, most Chinese speech arrest areas were located at specific language production sites, which 50% positive sites in the ventral precentral gyrus, 28% in the pars opercularis and pars triangularis. Additionally, the left middle frontal gyrus (Brodmann’s areas 6/9) was found to be unique for Chinese production. Moreover, Chinese speakers’ inferior ventral precentral gyrus (Brodmann’s area 6) was used more often than English speakers (30). Therefore, the combinational speech arrest map can be divided into four clusters: Cluster 1 was mainly located in the ventral precentral gyrus and the pars opercularis, which contained the peak of speech arrest in the ventral precentral gyrus; Cluster 2 was in the ventral and dorsal precentral gyrus; Cluster 3 was in the supplementary motor area; Cluster 4 was in the posterior superior temporal gyrus and supramarginal gyrus (31).

The white matter tracts transmit information between different cortical regions, and their connectivity enables the central nervous system to function normally. Hence, the idea of “eloquent areas” should be expanded to include deep structures rather than a purely cortical concept. BOLD-fMRI and DTI tractography have been routinely applied in many neurosurgical procedures for cortical eloquence and white matter assessment.

However, conventional DTI tractography methods rely on the technician to manually select the region of interest based on prior knowledge of anatomy, then generate the fibers from there, then clean and refine the bundle obtained. This approach is time-consuming, prone to operator-induced deviation, and without any “gold standard” (32). Therefore, the training of a specialized technician does need a learning curve. Unfortunately, most hospitals in China do not even provide this technician position due to the huge inequalty of medical resources among different neurosurgery centers (33). Although there are several automatic algorithms for whole-brain tractography, tracts derived from these algorithms can deviate significantly from each other, making it difficult to identify the most accurate one (12). A feasible automatic bundle-specific neurofiber tractography algorithm is not yet available.

Given this, we have developed an automatic bundle-specific white matter fiber tracking tool (DiffusionGo) with a fully automatic multiple assigned criteria (MAC) algorithm for bundle-specific neurofiber reconstruction (14) to achieve multimodality modeling and visualization for precision surgical planning with minimal human processing error. Previous bundle-specific tractography studies mostly used manually tractography but faced significant challenges in identifying accurate tracts, especially in cases with apparent lesion effects for neurosurgical implementation (34–36). In contrast, DiffusionGo was developed based on anatomical connectivity from clinical and autopsy data.

Modern neuroscience research has revealed a dorsal language pathway (arcuate fasciculus, AF, and superior longitudinal fasciculus II, SLF-II) using DTI tractography responsible for verbal repetition by integrating sensory-motor information. The SLF/AF system is the most comprehensive association fiber system at the lateral surface, connecting the frontal, temporal, parietal, and occipital lobes. AF was considered to connect the classic Broca’s area and Wernicke’s area. Specifically, the SLF connects the frontal and parietal lobes, allowing communication between the dorsal premotor and prefrontal cortices to the angular gyrus. The SLF also contains frontal-to-parietal connections terminating within the supramarginal gyrus (26).

In these three cases, both AF and SLF were automatically generated using DiffusionGo. In Case 1, the actual speech arrest areas were located at the tract-based cortical termination of SLF-II. Independently, our glioma team’s study of language mapping in glioma surgery showed that in Chinese people the goodness of fit between the terminal territories of the manually tracked AF and SLF and the DSC-mapped eloquent speech output area in the pars opercularis and ventral premotor cortex was 82% and 86%, respectively (25). For Case 2 treated in another institute, although nonawake surgery was performed, the automatic tracked neurofibers still augmented surgical plans, with an exceptionally safe approach design, for removing highly invasive gliomas by analyzing the relationship between the tumor and white matter tracts. Similar results were reported previously (37) and as in Case 3.

The major limitation of this pilot study was its limited sample size and lack of a control group. However, these results did show the promising value of the clinical implementation of DiffusionGO. We are now pursuing a multicenter clinical trial of this automatic DTI tractography pipeline to prove its potential role as an efficient, clinically applicable bundle-specific tractography tool to augment technical equality and improve surgical planning precision across different hospitals in China.

## 5 Conclusion

We demonstrated the application of DiffusionGo in three language-related cases. The fully automatic processing pipeline may provide the technician or surgeon with a solution to reduce time cost and operating error. We believe that this promising technique can improve care quality and surgical procedure quality across different facilities.

## Data availability statement

The raw data supporting the conclusions of this article will be made available by the authors, without undue reservation.

## Ethics statement

The studies involving human participants were reviewed and approved by IRB of Huashan Hospital, Fudan University (KY2021-452 and KY2019-008). The patients/participants provided their written informed consent to participate in this study. Written informed consent was obtained from the individual(s) for the publication of any potentially identifiable images or data included in this article.

## Author contributions

Conceptualization, JS and C-PL. Methodology, YY, TQ, SC and S-PC. Software, Y-HC, Y-CH, Y-TK and K-TK. Validation, GX, YY and ZY. Investigation, YY and TQ. Resources, WZ and C-PL. Data curation, YY and TQ. Writing—original draft preparation, YY and SC. Writing—review and editing, SC, JS and C-PL. Visualization, YY, GX, Y-TK and K-TK. Supervision, JS and C-PL. Funding acquisition, JS. All authors contributed to the article and approved the submitted version.
