# Supplementary material for: Automatic bundle-specific white matter fiber tracking tool using diffusion tensor imaging data: A pilot trial in the application of language-related glioma resection
Source: Front Oncol. 2023 Mar 24;13:1089923. doi: 10.3389/fonc.2023.1089923 (PMC10080097; doi:10.3389/fonc.2023.1089923)
Supplement: Supplementary file 1 [file DataSheet_1.pdf]

The validation of automatic fiber tractography (unpublished study)

This validation set included a total of 31 patients (20 men, 11 women). The mean age was 40.48 years, ranging from 20 to 66 years. Twenty patients were diagnosed with astrocytoma (17 WHO grade II and 3 WHO grade III). Six patients had GBM, 4 patients with oligodendrocytoma (2 WHO grade II and 2 WHO grade III), and a patient was diagnosed with WHO grade II pleomorphic xanthoastrocytoma. All tumors were located at left hemisphere, 8 (25.8%) were located at frontal lobe, 8 (25.8%) were located at parietal lobe, and 15 (48.4%) were located at temporal lobe.

A total of 97 DCS stimulus points finally recorded in 31 patients. However, by excluding the non-language-related stimulus DCS points, 90 DCS points were remaining. 84 were speech arrest, and 6 were anomia. Thirty-nine (43.44%) of DCS points were targeted on AF, 72 (80%) of DCS points were targeted on SLF-II, and 77 (85.55%) of DCS points were targeted on AF and SLF-II. Among these 77 DCS points, 5 were only targeted on AF, 37 were targeted on SLF-II, and 35 were both targeted on AF and SLF-II.

This study demonstrated that the fully automatic processing pipeline may provide the technician or surgeon with a solution to reduce time cost and operating error. We believe that this promising technique can improve care quality and surgical procedure quality across different facilities.

Table S1. DiffusionGo processing time with all tracts reconstructed.

Case 1			
seed count	1M	500K	50K
time elapsed	55:12	43:6	31:45
Case 2			
seed count	1M	500K	50K
time elapsed	51:36	42:57	31:45
