# Supplementary material for: Automatic bundle-specific white matter fiber tracking tool using diffusion tensor imaging data: A pilot trial in the application of language-related glioma resection
Source: Front Oncol. 2023 Mar 24;13:1089923. doi: 10.3389/fonc.2023.1089923 (PMC10080097; doi:10.3389/fonc.2023.1089923)
Supplement: Supplementary file 2 [file DataSheet_2.pdf]

The case was recently conducted at Huashan Hospital of Fudan University. This 35-year-old female suffered from intermediate headache for half year. Conventional MR indicates a left parietal lesion of 2 cm, with high signal in T2 Flair and not enhanced after contrast. Preoperative DTI showed the AF and SLF were located below and ahead of the tumor, respectively. The lesion and fiber were tracked by two different neurosurgeons (Figure S1B and Figure S1C), and the variability of results was partly due to operator experiences and personal tendency.

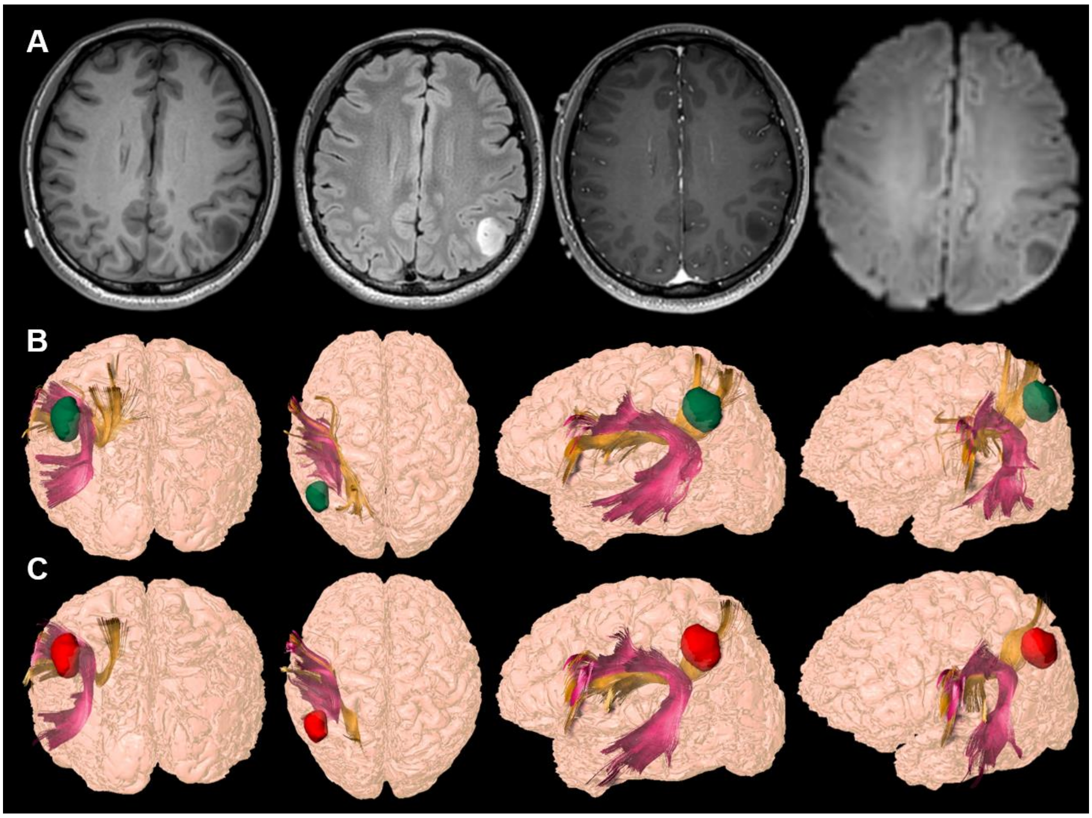

Figure S1: exemplary imaging of a patient with left parietal lobe astrocytoma.
Conventional MR of the lesion was shown in A (from left to right: T1W, T2 Flair, T1 Gd-enhanced and DWI). The relative spatial position of AF (pink), SLF (Yellow) and tumor was demonstrated in B (the first neurosurgeon) and C (the second neurosurgeon).
